# Gene Expression Classifier Reveals Prognostic Osteosarcoma Microenvironment Molecular Subtypes

**DOI:** 10.3389/fimmu.2021.623762

**Published:** 2021-04-20

**Authors:** Yi-Jiang Song, Yanyang Xu, Chuangzhong Deng, Xiaojun Zhu, Jianchang Fu, Hongmin Chen, Jinchang Lu, Huaiyuan Xu, Guohui Song, Qinglian Tang, Jin Wang

**Affiliations:** ^1^ Department of Musculoskeletal Oncology, Sun Yat-sen University Cancer Center, Guangzhou, China; ^2^ State Key Laboratory of Oncology in Southern China, Collaborative Innovation Center of Cancer Medicine, Guangzhou, China; ^3^ Department of Pathology, Sun Yat-sen University Cancer Center, Guangzhou, China

**Keywords:** osteosarcoma, tumor immune microenvironment, molecular subtyping, tumor-infiltrating lymphocytes, gene expression classifier

## Abstract

Osteosarcoma (OSA) is the most common bone malignancy and displays high heterogeneity of molecular phenotypes. This study aimed to characterize the molecular features of OSA by developing a classification system based on the gene expression profile of the tumor microenvironment. Integrative analysis was performed using specimens and clinical information for OSA patients from the TARGET program. Using a matrix factorization method, we identified two molecular subtypes significantly associated with prognosis, S1 (infiltration type) and S2 (escape type). Both subtypes displayed unique features of functional significance features and cellular infiltration characteristics. We determined that immune and stromal infiltrates were abundant in subtype S1 compare to that in subtype S2. Furthermore, higher expression of immune checkpoint PDCD1LG2 and HAVCR2 was associated with improved prognosis, while a preferable chemotherapeutic response was associated with FAP-positive fibroblasts in subtype S1. Alternatively, subtype S2 is characterized by a lack of effective cytotoxic responses and loss of major histocompatibility complex class I molecule expression. A gene classifier was ultimately generated to enable OSA classification and the results were confirmed using the GSE21257 validation set. Correlations between the percentage of fibroblasts and/or fibrosis and CD8^+^ cells, and their clinical responses to chemotherapy were assessed and verified based on 47 OSA primary tumors. This study established a new OSA classification system for stratifying OSA patient risk, thereby further defining the genetic diversity of OSA and allowing for improved efficiency of personalized therapy.

## Introduction

Osteosarcoma (OSA) is the most common primary bone malignancy, yet therapeutic strategies have improved little over the past decades (1). Comprehensive analysis by the Pan-Cancer Analysis of Whole Genomes (PCAWG) found that OSA has a low tumor mutation burden but exhibits high-confidence chromothripsis (2, 3). Our previous study also confirmed that OSA has a high number of genomic structural variations (4), making most targeted drugs designed for driver mutations not suitable. Therefore, despite immune or targeted therapies being widely used in various cancers, the current standard treatment for OSA is chemotherapy combined with surgery (5). With the development of tumor research into the “Precision medicine omics era” (6), there is an urgent need to develop new OSA molecular subtyping systems to find effective treatment strategies.

The immune contexture and immunoscore of the tumor microenvironment (TME) can be used to evaluate and predict treatment response and drug resistance (7). Tumor-infiltrating lymphocytes (TILs), major histocompatibility complex (MHC) class I molecules, and immune checkpoints inhibitors are associated with a survival benefit for patients with malignancies, and are related to immunotherapy and chemotherapy responses (8, 9). Meanwhile, tumors can be classified into different groups based on their programmed death-ligand 1/2 (PD-L1/PD-L2) status, the presence or absence of TILs with PD-L1/PD-L2, and the presence of antigen-specific CD8⁺ TILs that drive adaptive immune resistance, referred to as a “hot tumor” (10). Examination of OSA biopsy specimens for the evaluation of clinical outcomes has revealed that high expression of T cell activation markers prior to administration of chemotherapy was associated with good prognosis (11). Furthermore, we previously reported that a coordinated T-helper type 1 (Th1) cell and cytotoxic immune infiltration in OSA tumors is associated with a favorable clinical outcome in terms of chemotherapy response and overall survival (12, 13). Molecular subtyping of OSA and its complex interactions between clinical response and TME need to be elucidated.

Increasing evidence indicates that stromal cells surround tumors and establish a dependency relationship with TILs, which have critical roles in tumor progression and metastasis (14). In addition, stromal cells are believed to serve as a means for activating the local immune response to “heat up” a tumor (15). Importantly, tumor-associated fibroblasts (TAFs) can also become activated, leading to recruitment of immune cells and regulation of tumor immunity in the TME (16, 17). Previously, it was widely believed that TAFs or fibrosis promoted tumor progression; however, recent studies have suggested alternate views and identified a TAF subtype termed tumor‐restraining fibroblasts (18, 19). The presence of fewer myofibroblasts in tumors is associated with poor survival in both mice and human patients (20). Meanwhile, the expression of chemokine CXCL12 is strongly correlated with overall survival in OSA (21), and targeting CXCL12 derived from FAP-positive TAFs enhances the sensitivity of tumors to immunotherapy (22). On the other hand, analysis of how TAFs affect clinical outcomes has often yielded contradictory results (23). Therefore, considering immune or stromal cells independently in the TME may limit the beneficial effects of newly developed therapeutic strategies.

To accelerate the clinical application of molecular subtyping in OSA, we plan to further explore optimization of the gene classifier for use in precision medicine by advancing the current understanding regarding the genetic mechanisms underpinning its complex regulatory network. In this study, we describe a molecular classification of the OSA microenvironment, along with the molecular features and clinical characteristics of its subtypes. We emphasize that consideration of both infiltrating immune and stromal cells is required to obtain an effective classifier for personalized medicine.

## Materials and Methods

### Processing of OSA Gene Expression Data

The results published here as a training set are based on data generated by the OSA project of the Therapeutically Applicable Research to Generate Effective Treatments program (TARGET, https://ocg.cancer.gov/programs/target). Level three RNA-Seq data and relevant clinical information for OSA patients were downloaded from the TARGET OSA project. We used normalized Transcripts Per Kilobase Million (TPM) values of 92 samples for gene expression analyses. To prevent errors, TPM values were further log2-transformed using an offset of 1 in some of the analyses. The Gene Expression Omnibus (GEO) dataset GSE21257 was used as a validation set and included 54 gene expression profiles of pre-chemotherapy biopsies from patients with OSA (24). The mRNA data and survival information of dataset GSE21257 were downloaded from the GEO database.

### Expression Data Grouping and Difference Analysis

Based on the clinical information of each sample, we further collated the annotated data and divided it into groups (**Table S1**). Differential analysis of each group and subtype was performed using R package *limma*. First, we divided survival events into three groups according to the time of overall survival: Good (≥5 years), Poor (<2 years), and Medi (≥2 and <5 years). In many studies that evaluated the prognosis of OSA, patients with a tumor necrosis rate greater than 90% after chemotherapy (grade 3 or 4 in the Huvos system) were reported to have a better overall survival than that of other groups with lower necrosis rates (grade 1 or 2) (25, 26). Excluding samples lacking histological annotation, we classified chemotherapeutic responses as Good and Poor based on tumor necrosis rates or the Huvos system.

### Gene Enrichment and Survival Analysis

Pathway analysis was performed on ranked data from differential analysis using the single sample Gene Set Variation Analysis (GSVA) algorithm in the *GSVA* package (27) and reference gene sets from the Molecular Signatures Database (MSigDB, version 7.1) of the KEGG pathway 186 gene sets (c2.cp.kegg.symbols.gmt). Enrichment results satisfying a nominal significance with a p-value <0.05 were considered statistically significant. We used package *clusterProfiler* to perform enrichment analysis with Gene Ontology Biological Process (GO BP) terms associated with significant genes (28). Kaplan-Meier survival analysis and visualization were performed using the packages *survival* and *survminer* in R. The package *pROC* was used to display and analyze receiver operating characteristic (ROC) curves.

### Identification of Molecular Subtypes Based on Expression Patterns

Gene expression data were screened for prognostic genes (p < 0.05 and fold-change >1.5) of overall survival events (Good *vs.* Poor) for OSA patients. To identify expression-dependent subgroups, we performed nonnegative matrix factorization (NMF) on the expression matrix of prognostic-related genes using package *nmf*. We applied the unsupervised non-smooth NMF algorithm to reduce dimensionality and obtain coefficient and basis matrices (29). The hierarchical clustering method provided by the R function *hclust* was used to verify molecular typing. Principal Component Analysis (PCA) was applied to extract the expression pattern of genes and transform them into new features to then determine group variation using packages *FactoMineR* and *factoextra*.

### Estimation of OSA TME Cell Infiltration

The R package *ESTIMATE* was used to provide estimated scores of tumor purity and to evaluate the overall level of immune cell infiltration in tumor tissues (30). Enrichment of 24 immune cell types was computed through gene set variant analysis using the Immune Cell Abundance Identifier (ImmuCellAI) (31), which was applied to estimate differences in immune cell infiltration of the various groups. Furthermore, the webtool *xCell* was intended to perform cell type enrichment analysis for 14 stromal cell types (32).

### Signature Selection and Validation Through Classifier

For validation of the molecular subtyping, we conducted a classification analysis including all genes differentially expressed between the subtypes as the training set based on the nearest shrunken centroids method using package *PAMR* (33). We then carried out 10-fold cross-validation and divided the data for all genes belonging to the signature into the TARGET training set.

### Patients and Tissue Samples

Primary surgical tumor samples were collected from 47 patients with OSA (median age 15 years, range: 7–38) at Sun Yat-sen University Cancer Center, from November 2017 to October 2019. The definite diagnosis was confirmed histopathologically by two experienced pathologists. All patients underwent four to six cycles of standard neoadjuvant chemotherapy with the MAPI regimen (Methotrexate 8–12 g/m^2^, Ifosfamide 12–15 g/m^2^, and Doxorubicin 75–90 mg/m^2^ + Cis-platinum 80–120 mg/m^2^) following surgical resection according to the NCCN guideline. The clinicopathological characteristics of the 47 patients are listed in **Supplementary Table S2**. RECIST1.1 criteria were used to estimate the curative effect of neoadjuvant chemotherapy mainly based on the change in tumor volume according to MRI of the primary site and the development of metastatic lesions according to chest CT, as previously reported (34, 35). This research was approved by the Institutional Ethics Committee of Sun Yat-sen University Cancer Center.

### H&E and Immunohistochemical Staining

Hematoxylin & Eosin staining was carried out on 5-μm sections of formalin-fixed, paraffin-embedded (FFPE) OSA tissues obtained with a slicer system (Ventana Discovery XT automated system, Ventana Medical Systems, Tucson, AZ, USA). Briefly, slides were overstained with hematoxylin for 5 min, differentiated and de-stained with acidic alcohol, rinsed, and blued with bicarbonate. Finally, sections were dehydrated and stained with eosin.

For immunohistochemistry (IHC), tissue slides from paraffin-embedded tissues were dewaxed using xylene, hydrated in different gradient ethanol, and then subjected to citrate buffer-induced antigenic epitope retrieval. Goat serum buffer (ZSGB-BIO, Beijing, China) was applied to the sections on the slides for non-specific antigenic blocking. The sections were then incubated with primary antibody CD8 (1:100, ZSGB-BIO, #ZA-0508) overnight at 4°C. After incubating with secondary antibodies (ZSGB-BIO), DAB substrate solution was applied to reveal the color of the antibody staining and hematoxylin was counterstained on the slides by immersing their sides.

### Immunoreactive Score

The final immunohistochemical scores were evaluated and recorded by two independent pathologists under double-blind conditions. Tumor cell percentages and histologic fibroblast and/or fibrosis extent were estimated in hematoxylin–eosin (H&E)-stained slides according to the median fraction of tumor cell nuclei and the percentage of fibrotic-like cells presented in each FFPE tissue section. The number of CD8-positive cells was counted in 10 random power fields (400×; high-power field, HPF) per section in microscopes. The averages of the scores above were calculated for statistical analysis.

### Masson’s Trichome and Fibrosis Quantification

FFPE OSA specimens were sectioned at 5-μm thickness for Masson’s trichrome staining. Microscopy images were obtained under five random fields (100×) in a blinded fashion and analyzed using Image J software (http://rsbweb.nih.gov/ij/) for collagen fibers. Fibrosis areas were measured and expressed as percentage of the entire area of tumor tissue by visualizing the blue-stained areas that colocalized in the tumor area. Blue-stained areas and non-stained areas from each section were determined using color-based thresholding in ImageJ software automatically. As described previously (36), the percentage of fibrosis area was calculated as the summed positive areas divided by the area of the total region of interest.

### Statistical Analysis

All statistical analyses and data visualization were performed using R v3.5.2 and Prism v5 (GraphPad Software, San Diego, CA, USA). To compare the correlation between genes, we calculated the Spearman rank correlation coefficient of gene expression. Statistical differences between the two groups were calculated using two-tailed *t-*test with Mann-Whitney U test. For correlation and difference analyses, a threshold p-value <0.05 was considered statistically significant. The significance of comparisons was based on the Wilcoxon rank sum test, and are shown as mean p-values (*p < 0.05; **p < 0.01).

## Results

### Distinct Molecular Subclasses Defined by Gene Expression Features are Linked to Prognosis

**Figure 1:**
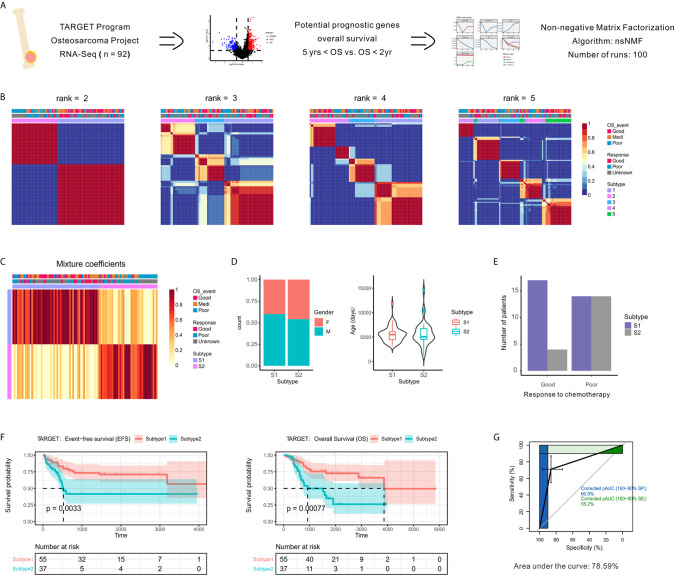
Identification of prognostic molecule subtypes in the OSA tumor microenvironment. **(A)** Schematic flow diagram of the analytic procedures used for cluster analysis. **(B)** Nonnegative matrix factorization (NMF) classification with rank K from 2 to 5 identifies bases consistent with the prognostic significance subgroups. **(C)** Heatmap of the metagene expression profiles matrix. **(D)** Patient characteristics between two subtypes of patients (S1 and S2). **(E)** Number of patients with different chemotherapy responses between the two subtypes. **(F)** Kaplan-Meier event-free survival and overall survival curves for patients with OSA demonstrating significant differences in survival when separated into subgroups. **(G)** Receiver operating characteristic curve of subtypes.

To identify genetic events underlying the development and progression of OSA, we conducted an integration analysis of gene expression profiles using a collection of outcome-annotated clinical specimens. Attesting to the molecular heterogeneity of OSA, unsupervised classification using NMF designed for expression profile analysis revealed distinct transcriptomic subtypes. A schematic flow chart of our study used to identify the OSA subclasses is shown in **Figure 1A**, and clinical characteristics of the patients are shown in **Table S1**. A set of 620 prognostic differentially expressed genes (DEGs) was identified as being a characteristic gene signature for development of the OSA molecular subtypes.

To identify unsupervised classification of distinct patient subgroups, we applied NMF clustering analysis to the expression matrix of prognostic DEGs. The clusters showed a nested structure as k increased from 2 to 5 and the consensus was strong for k = 2 (**Figures 1B, C**). The unsupervised clustering revealed two primary functional associations that acted as different subtypes of the OSA TME (S1 and S2). As shown in **Figure 1D**, no association between subtype and gender or age was found. Histological responses to neoadjuvant chemotherapy were also classified as Good or Poor responders based on the percentage of tumor necrosis (**Table S1**). Interestingly, better chemotherapy response was observed in S1 compared to S2 (**Figure 1E**).

As chemotherapy responses could be used to indicate survival status, we also found a consistent survival bias between subtypes (**Figure 1F**). The gene expression patterns that characterized patients with OSA TME subtype S1 and significantly prolonged disease-free and overall survival differed from those that characterized patients with OSA TME subtype S2 and unfavorable outcomes. As shown in **Figure 1G**, the overall classification accuracy far exceeded the accuracy of few gene signatures, with the area under curve reaching 78.59%. Therefore, the unsupervised analysis of gene expression profiles performed revealed two distinct OSA microenvironment subtypes.

### Transcriptomic Profiling Reveals Distinct Immune Microenvironment Characteristics

**Figure 2:**
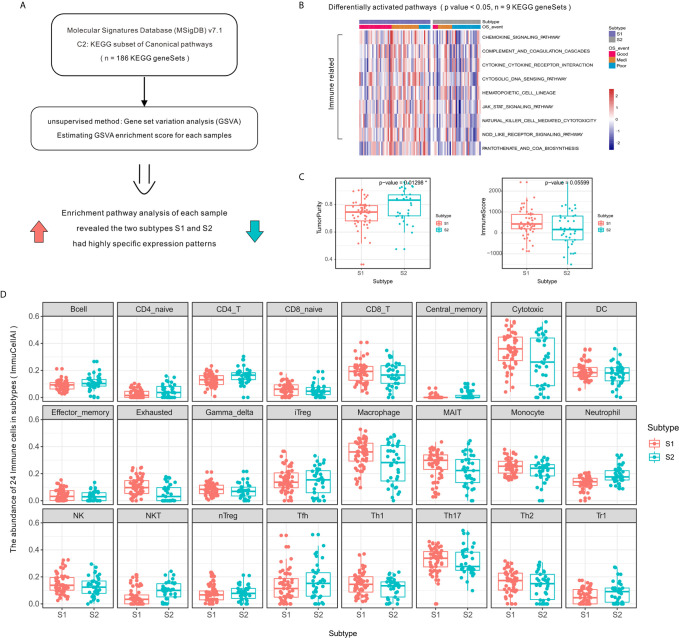
Activation level of specific immune-related pathways and increased immune cell infiltration in OSA TME subtype S1 compared to that of subtype S2. **(A)** Schematic diagram of enrichment analysis of signaling pathways. **(B)** Heatmap showing enrichment scores of significantly different KEGG gene sets. **(C)** Tumor purity and ImmuneScore for each subtype generated using the ESTIMATE algorithm. **(D)** Immune cell infiltration differences between OSA TME subtypes using ImmuCellAI. *mean p-values < 0.05.

To elucidate the signaling pathways affected by different TME subtypes, we performed KEGG pathway enrichment analysis using the GSVA algorithm (**Figure 2A**). Enrichment pathway analysis of each sample revealed the two subtypes had specific expression patterns. Several immune pathways were enriched in subtype S1 (**Figure 2B**), the subtype associated with a better clinical benefit response.

The high level of immune cell infiltration in subtype S1 corresponded to a proportionally lower tumor content (**Figure 2C**), as computationally predicted using the ESTIMATE algorithm. To compare the transcriptional profiles of immune cell subpopulations, we evaluated the abundance of 24 immune cell types in OSA using ImmuCellAI and found a higher abundance of CD8⁺ T, Cytotoxic, and Th1 cells in patients defined as subtype S1 (**Figure 2D**); meanwhile a higher abundance of Th2 cells was observed for subtype S2. There was, therefore, a need to verify the observed differences using specific immune cell markers, which was achieved in subsequent experiments.

### Composition and Molecular Features of Infiltrating Immune Cells of OSA TME Subtypes

To investigate the relevance of the infiltrating immune cells in the identified TME subtypes, we used the following immune-related markers: CD8A, CD3D, protein tyrosine phosphatase, receptor type, C (PTPRC; CD45), granulysin (GNLY), and granzyme K (GZMK) related to activated CD8⁺ cytotoxic T-cell lymphocytes (37); MHC class I molecules including beta-2-microglobulin (B2M) and human leukocyte antigen A–C (HLA-A/B/C); interferon gamma receptor 2 (IFNGR2) for Th1-type CD4⁺ T-cells and signal transducer and activator of transcription 6 (STAT6) for Th2-type CD4⁺ T-cells (CD4); the immune checkpoint markers, programmed cell death 1 (PDCD1; PD-1), CD274 (PD-L1), programmed cell death 1 ligand 2 (PDCD1LG2; PD-L2), cytotoxic T-lymphocyte-associated protein 4 (CTLA4), lymphocyte-activation gene 3 (LAG3), hepatitis A virus cellular receptor 2 (HAVCR2; TIM-3), and T-cell immunoreceptor with Ig and ITIM domains (TIGIT) used as targets for immunotherapy.

**Figure 3:**
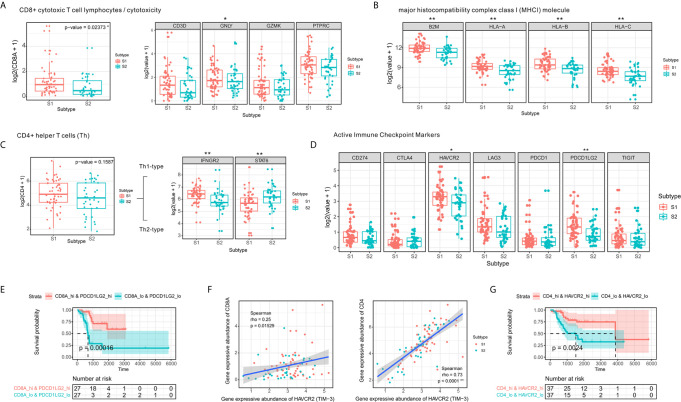
CD4⁺ Th1 and CD8⁺ cytotoxic T-cell infiltration and increased expression of immune checkpoint molecules. **(A)** Comparison of expression levels of CD8⁺ T-cell markers. **(B)** Comparison of expression levels of MHC class I molecules. **(C)** Boxplot of the expression of CD4⁺ T-cell markers in two the OSA TME subtypes S1 and S2. **(D)** Comparison of levels of immune checkpoints in the different subtypes **(E)** Prognostic value of CD8A+PDCD1LG2 co-expression groups based on Kaplan-Meier survival analysis. **(F)** Spearman correlation between HAVCR2 and CD4/CD8A expression. **(G)** Prognostic value of CD4+HAVCR2 co-expression groups based on Kaplan-Meier survival analysis. The significance levels of the p-values are presented as *p < 0.05 or **p < 0.01.

Overcoming the failure of tumor immunosurveillance largely requires sufficient activation of MHC class I molecule presenting antigens to CD8⁺ cytotoxic T lymphocytes. Here we observed increased expression of CD8⁺ cytotoxic T-cell genes and increased abundance of MHC class I molecules in subtype S1 (**Figures 3A, B**). Moreover, the differential expression analysis showed that CD4⁺ T-helper cells in the two subtypes were polarized toward Th1 and Th2 phenotypes, respectively (**Figure 3C**). Among the immune checkpoints, we found that HAVCR2, and PDCD1LG2 were highly expressed in the S1 subtype compared to S2 (**Figure 3D**).

A strong correlation has been established between checkpoints and infiltrating lymphocytes and patient survival (38). We observed similar results in the co-expression survival analysis of PDCD1LG2 with CD8⁺ T-cells (**Figure 3E**). As HAVCR2 was initially identified as a receptor expressed on interferon-γ-producing CD4⁺ Th1 cells and CD8⁺ cytotoxic T-cells (39), we performed Spearman correlation analysis for HAVCR2 and CD4/CD8A expression in OSA TME (**Figure 3F**) and observed a strong correlation between HAVCR2 and CD4 expressions. Meanwhile, survival analysis suggested that HAVCR2 and CD4 co-expression was statistically significantly associated with overall survival of OSA patients (**Figure 3G**). Overall, the genes highly expressed in subtype S1 were primarily associated with CD4⁺ Th1 cells, CD8⁺ cytotoxic T-cells, and immune checkpoints. In contrast, patients with subtype S2 exhibited a different immune pattern which inhibited overall expression of MHC class I molecules, higher activity of Th2 cells and a poor prognosis.

### Significant Differences Observed Among Subtypes of Stromal Cells in Microenvironment

**Figure 4:**
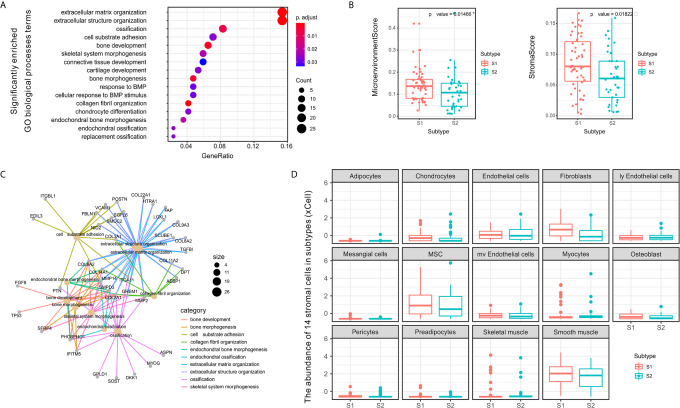
Differences between S1 and S2 subtypes related to extracellular matrix and stromal cells in the tumor microenvironment. **(A)** Dot plot showing the number of genes associated with the GO BP terms (circle size) and the p-adjusted values for these terms (circle color) based on the DEG enrichment results between the two subtypes S1 and S2. **(B)** Category net plot showing the relationships between genes associated with the top 10 most significant GO BP terms. **(C)** Evaluation of the infiltration scores for the entire microenvironment and stromal cells between the S1 and S2 TME subtypes. **(D)** Boxplot displaying enrichment scores for 14 stromal cell types determined using xCell across the two subtypes. The significance levels of the p-values are presented as *p < 0.05.

To obtain a clearer understanding of the biological processes associated with the enriched gene expression variants, we evaluated the enrichment of DEGs between subtypes according to GO BP terms. We found that the enrichment process was closely associated with matrix or stromal cells in the microenvironment (**Figures 4A, B**).

To evaluate the microenvironment characteristics between S1 and S2, we estimated microenvironment and stromal scores using *xCell* and found that subtype S1 was characterized by greater cell infiltration in the microenvironment, particularly regarding stromal cells, which was significantly higher than that observed for subtype S2 (**Figure 4C**). Notably, we also compared the abundance of 14 types of stromal cells in the two TME subtypes and found significant differences in the enrichment of fibroblasts (**Figure 4D**). Previous studies have demonstrated that fibroblasts can be divided into different subtypes according to function based on whether tumor progression is promoted or suppressed (40). Therefore, in subsequent experiments we further evaluated the molecular features of the stromal fibroblasts in the OSA TME subtypes.

### Heterogeneity of Fibroblast Types in OSA TME S1 and S2 Subtypes

**Figure 5:**
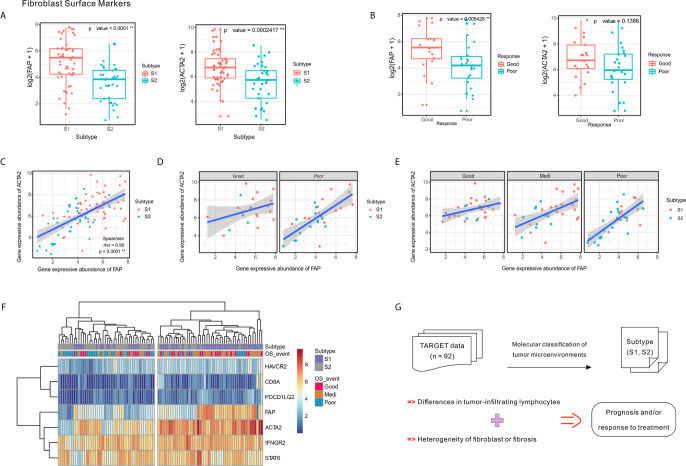
Expression of fibroblast markers FAP and ACTA2 and their correlation with clinical response and tumor subtypes. **(A)** Expression levels of FAP and ACTA2 in OSA TME subtypes S1 and S2. **(B)** Expression levels of FAP and ACTA2 in different chemotherapeutic response events. **(C–E)** Spearman correlation between FAP and ACTA2 expression in whole samples, as well as different prognoses and chemotherapy responses. **(F)** Heatmap of FAP/ACTA2 and immune-related marker expression levels. **(G)** Schematic of TME heterogeneity in OSA subtypes S1 and S2. The significance levels of the p-values are presented as **p < 0.01.

To confirm the fibroblast heterogeneity in the OSA TME, we used fibroblast activation protein alpha (FAP) and actin alpha-2 (ACTA2; alpha-smooth muscle actin, α-SMA) as markers of fibroblasts. The fibroblast abundance was verified in multiple OSA specimens and the relative abundance of FAP- and ACTA2-positive cells was confirmed to be substantially higher in subtype S1 compared to S2 (**Figure 5A**).

Comparing specimens from groups of patients with differing chemotherapy responses, we found that greater levels of FAP expression was associated with higher tumor necrosis rates; however, significant differences were not observed regarding ACTA2 expression levels (**Figure 5B**). When considering chemotherapy response and prognosis relative to FAP and ACTA2 expression profiles, the correlation between them changed, suggesting that different fibroblast subsets may dominate in different statements (**Figures 5C–E**).

Since the TME includes a combination of immune and stromal cells, we also evaluated the correlation between FAP/ACTA2 expression and immune-related markers. The differential expression of the immune and stromal markers indicated that patients could be divided into two groups, which were consistent with the TME molecular subtyping (**Figure 5F**). A schematic summary of the S1 and S2 subtype characteristics is shown in **Figure 5G**. Taking this data together we were able to classify the OSA TME into two subtypes based on molecular characteristics.

### Gene Expression Classifier for OSA TME Subtypes S1 and S2 was Validated Using Parallel Data

We also sought to generate a gene expression classifier for dividing OSA into prognostic TME subtypes and to confirm the preceding subtype-associated features. Histologic alterations are associated with treatment response. Specifically, higher degrees of necrosis (>90%) and fibrosis (>50%) in OSA resection specimens significantly correlate with better overall survival (41). To determine whether the molecular prognostic classifiers performed independently of chemotherapeutic factors, a series of pre-chemotherapy biopsy specimens were evaluated using the GSE21257 validation set (24).

**Figure 6:**
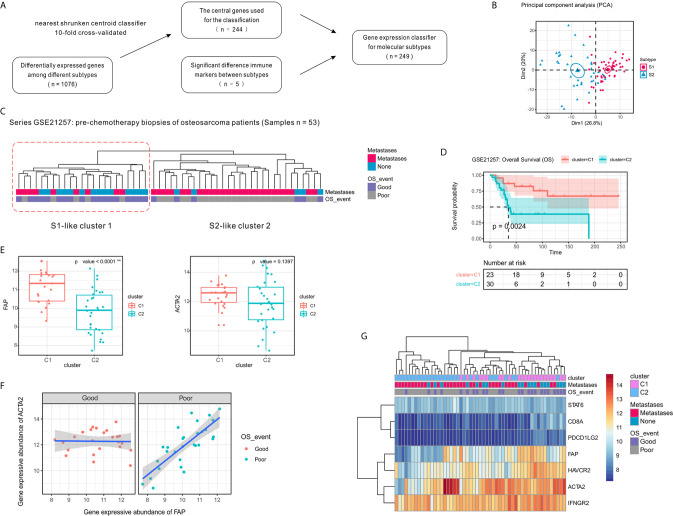
Confirmation of the molecular classification of the OSA tumor microenvironment using GEO dataset GSE21257. **(A)** Flow diagram for signature selection and construction of the molecular classifier. **(B)** PCA for the clustering genes of the classifier with OSA TME subtypes S1 and S2. **(C)** Clustering of patients with OSA in the GSE21257 dataset using unsupervised analysis of the gene expression classifier. **(D)** Kaplan-Meier overall survival curves for patients with OSA in the GSE21257 dataset when sorted into two clusters. **(E)** FAP and ACTA2 expression levels in the different clusters. **(F)** Spearman correlation between FAP and ACTA2 expression in different prognosis groups. **(G)** Heatmap of FAP/ACTA2 and immune-related marker expression levels. The significance levels of the p-values are presented as **p < 0.01.

To validate our two-subtype OSA TME classification, a 244-gene centroid classifier was developed from the discovery set using a 10-fold cross-validation approach. We found that by adding the five previously identified immune signatures (CD8A, IFNGR2, STAT6, HAVCR2, and PDCD1LG2), the S1 and S2 subtypes could be more effectively distinguished (**Figures 6A, B**). Due to annotation differences of the platforms, the classifier constructed using 249 genes was not able to obtain the entire expression matrix corresponding to the genes in the GSE21257 dataset. Based on the gene expression classifier using 199 of the 249 genes, clustering resulted in a dendrogram that separated the individual patients into two main clusters, one of which comprised patients that had better survival, similar to subtype S1 in the TARGET data, while the other appeared similar to subtype S2 (**Figure 6C**). To further validate the prognostic value of the two-subtype classification of OSA TME, we performed survival analyses and confirmed that the two clusters closely correlated with prognosis (**Figure 6D**).

Next, to determine whether the prognosis of patients with OSA correlated with fibroblast heterogeneity, we analyzed the expression of FAP and ACTA2 in relation to survival. Similar to the previously observed results, FAP was expressed at high levels in cluster C1 and was associated with better prognosis, while expression of ACTA2 was higher in cluster C1 with no significant effect (**Figure 6E**). Interestingly, we also observed that the correlation of FAP and ACTA2 expression with prognosis differed in the pre-chemotherapy samples of the GSE21257 dataset (**Figure 6F**). Fortunately, when we reduced the genetic signatures to seven as previous mentioned, we found that the expression patterns of the markers exhibited heterogeneity consistent with the molecular subtyping (**Figure 6G**).

### Fibroblasts and/or Fibrosis May Correlate With the Abundance of CD8^+^ Cells in OSA Tumors

Based on comprehensive analysis of gene expression profiles in TARGET and GSE21257, we developed a gene classifier that could distinguish patients with OSA according to their response to therapies. Interestingly, they presented two main gene types, immune genes represented by CD8, and matrix genes associated with fibroblasts and/or fibrosis.

**Figure 7:**
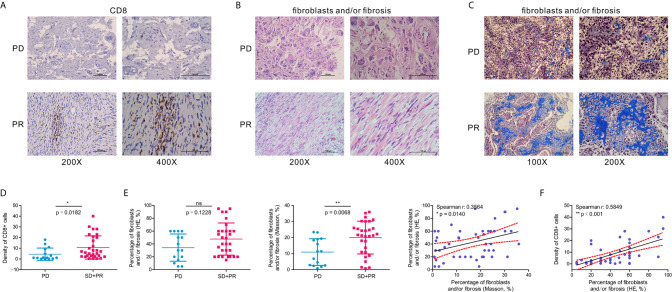
A higher percentage of fibroblasts and/or fibrosis correlates with the abundance of CD8^+^ cells in OSA tumors. **(A)** Immunohistochemistry for CD8 expression in OSA tumor tissues. **(B, C)** H&E staining and Masson’s Trichrome staining of fibroblasts and/or fibrosis in OSA tumors. **(D, E)** Comparison of the density per high-power field of CD8^+^ cells, and H&E/Masson’s Trichrome staining score of fibroblasts and/or fibrosis in non-responders (PD) and responders (PR and SD). **(F)** Correlation analyses of the abundance of CD8^+^ cells and fibroblasts and/or fibrosis. PD, progressive disease; PR, partial response; SD, stable disease. The significance levels of the p-values are presented as *p < 0.05, **p < 0.01 or ns, not significant p ≥ 0.05.

To verify our previous results, we selected 47 OSA clinical samples for histological verification. IHC staining results indicated that the presence of CD8^+^ cells is associated with a favorable response to chemotherapy (**Figures 7A, D**). At the same time, we also found that according to H&E and Masson’s Trichome staining of OSA tissues, a higher proportion of fibroblasts and/or fibrosis may be correlated with a better response after chemotherapy (**Figures 7B, C, E**). CD8^+^ T cell infiltration increased the percentage of fibroblasts and/or fibrosis, contributing to the anti-tumor efficacy of chemotherapy (**Figure 7F**).

Taken together, we were able to classify patients with OSA into two molecular subtypes based on expression pattern characteristics of the TME. These subtypes were associated with prognosis and chemotherapy response.

## Discussion

Based on the expression profiles of prognostic genes using unsupervised NMF-based classification, we stratified OSA patients and subdivided them into two distinct microenvironment-based subtypes, S1 (infiltration type) and S2 (escape type). Each subtype exhibited unique molecular characteristics and potential therapies with distinct clinical outcomes. While Wu et al. defined the immuno-genomic landscape of OSA and identified three immune clusters (42), our study lays the foundation for further studies examining how molecular characterization of the different subtypes could be used to guide individual immunotherapy. Therefore, in the current study we demonstrated the successful development and validation of a gene expression classifier useful for the prediction of prognosis specific for patients with OSA.

As TME heterogeneity contains multiple dimensions of information regarding patient prognosis and treatment response (43). Ogawa et al. identified three distinct stroma types (FAP/ACTA2-dominant fibroblast-rich stroma and collagen-rich stroma) in human pancreatic cancer (44), which were differentially associated with the immunosuppressive TME and patient outcomes. Our findings clearly demonstrate that combined consideration of immune and stromal factors is helpful for highlighting subtype characteristics. Specifically, the OSA TME subtype S1 had a gene expression pattern that indicated higher infiltration of immune and stromal cells was associated with better survival. Furthermore, the activation level of certain immune-related pathways and the expression of immune checkpoint molecules were also significantly higher in the S1 subtype, suggesting that such patients may benefit from immunotherapy. Furthermore, interactions of neoplastic cells with a diverse collection of immune and/or stromal cells along with extracellular matrix remodeling may influence tumor metastasis and therapeutic effects (45). As expected, we observed a strong positive enrichment of CD8 cytotoxic T cells and CD4 Th1 cells, which were associated with prognosis, along with high expression of immune markers and FAP fibroblast-related genes. Clinical sample results show that the infiltration abundance of immune cellular subset CD8^+^ cells were positively correlated with the percentage of fibroblasts and/or fibrosis, and prone to better prognosis and efficacy of neoadjuvant chemotherapy in OSA.

The failed OSA trial treatment studies have mainly selected PD-1 pathway inhibitors like SARC028 or PEMBROSARC instead of other checkpoint inhibitors (46, 47). Our previous results suggest that the degree of CD4^+^ Th1 cell infiltration is closely related to prognosis in OSA (13), and here we found that HAVCR2 was abundantly expressed in S1 subtype, strongly correlated with CD4 expression, and predicts a better prognosis. This suggests that targeting the HAVCR2 pathway might improve the efficacy of immunotherapy in OSA by activating CD4^+^ T cells and regulating Th1 cytokines.

Characteristic gene expression classifiers can be applied to distinguish different categories of patients at initial diagnosis or during the post-operation phase, and can be helpful in guiding the formulation and modification of treatment strategies for chemotherapy and immunotherapy (48). Our current study also found that the expression of fibroblast markers, FAP and ACTA2, correlated with clinical prognosis and chemotherapy response, suggesting the heterogeneity and versatility of TAFs in the OSA TME interacted with immune cells. Interestingly, FAP acts as a tumor suppressor in melanocytic cells that abrogates tumorigenicity through regulation of cell proliferation and survival (49). Of note, FAP is also used as a macrophage marker in TME (50), demonstrating the challenge associated with fully elucidating the specific significance of cell subsets in the OSA TME. Additional molecular studies are, therefore, required to confirm the function of the FAP^+^ ACTA2^+^ fibroblast subsets in the OSA TME.

**Figure 8:**
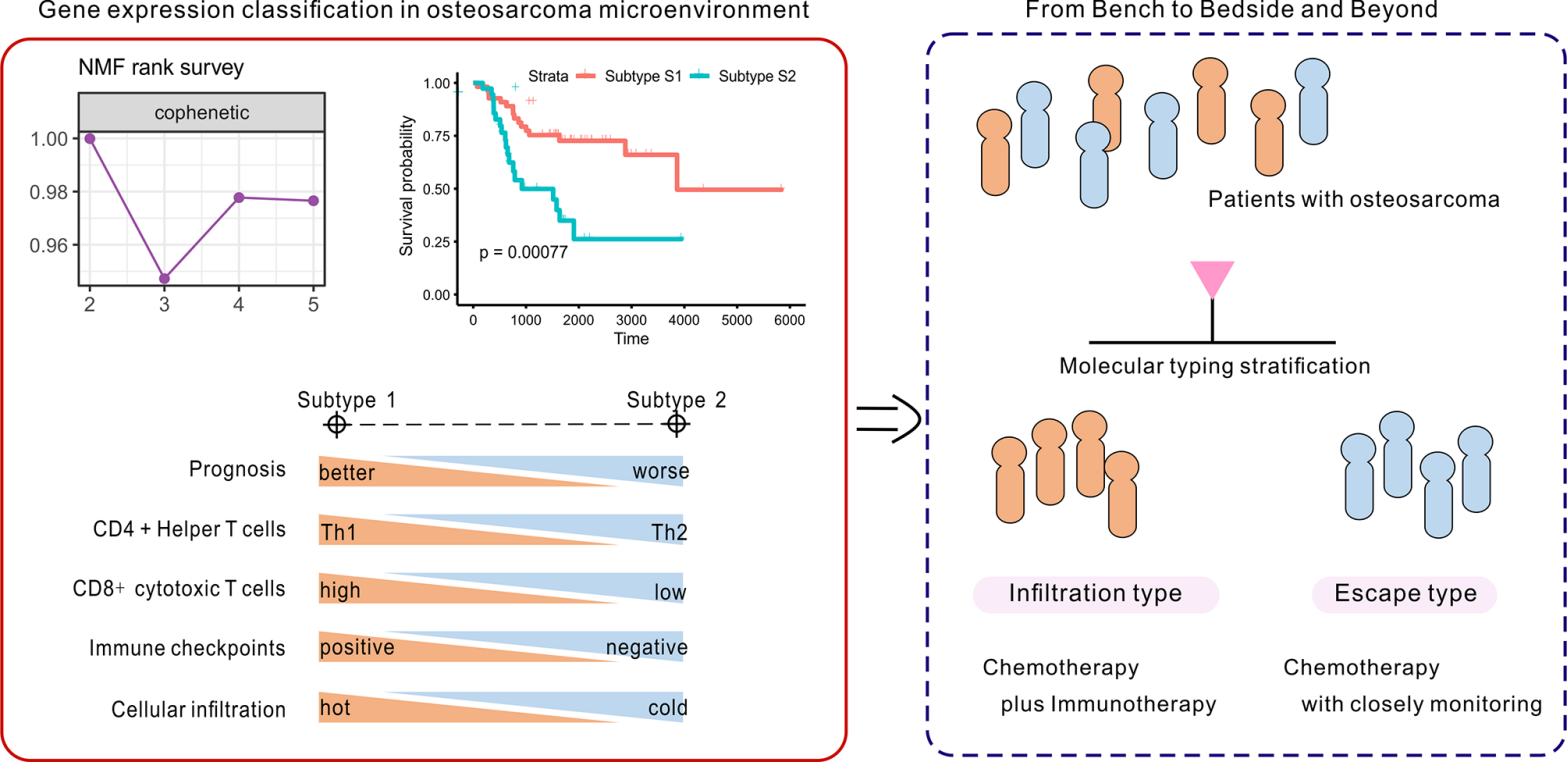
Identification and development of the gene-based classification system toward precision medicine in OSA.

In summary, patients with subtype 1 OSA tend to be associated with an infiltration type that resembles “hot” tumors, and thus combination chemo-immunotherapy may be considered to further improve patient outcomes (**Figure 8**). Alternatively, insufficient activation of MHC class I molecules served to restrict CD8 cytotoxic T cells to promote tumor immune escape in subtype S2 TME, to allow for proliferation of the tumor. While patients with OSA would be stratified by molecular typing toward precision medicine, closely monitoring and more aggressive treatment strategies are needed for patients with non-infiltration type. This novel molecular classification may prove beneficial in prognosing patients with OSA, predicting chemotherapy responses, and aiding in therapy decision making for different subgroups of patients with OSA.

## Data Availability Statement

Publicly available datasets were analyzed in this study. This data can be found here: Osteosarcoma project of the Therapeutically Applicable Research to Generate Effective Treatments program (TARGET, https://ocg.cancer.gov/programs/target). The Gene Expression Omnibus (GEO) dataset GSE21257 was downloaded from https://www.ncbi.nlm.nih.gov/geo/query/acc.cgi?acc=GSE21257.

## Ethics Statement

The studies involving human participants were reviewed and approved by the Medical Ethics Committee of the Sun Yat-Sen University Cancer Center. Written informed consent to participate in this study was provided by the participants’ legal guardian/next of kin.

## Author Contributions

Conceptualization and methodology, JW, QT, and YJS. Software, validation, formal analysis, investigation, resources, and data curation, YJS, YX, CD, XZ, JF, HC, HX, GS, and JL. Writing—original draft preparation, visualization, and writing—review and editing, YJS, QT, and YX. Supervision, project administration, and funding acquisition, JW, QT, JL, and YJS. All authors contributed to the article and approved the submitted version.

We would also like to thank all the members of the Bioinformatics Center in Sun Yat-Sen University Cancer Center, for their support, advice, and encouragement. Tissue samples applied in this work were provided by Department of Musculoskeletal Oncology in Sun Yat-Sen University Cancer Center, China.

## Funding

This research was funded by the National Science Foundation of China (81872183, 81872268, 91959115), Natural Science Foundation of Guangdong Province (2019A1515011192), and Postdoctoral Science Foundation of China (2019M653163).

## Conflict of Interest

The authors declare that the research was conducted in the absence of any commercial or financial relationships that could be construed as a potential conflict of interest.
