# Supplementary material for: Gene Expression Classifier Reveals Prognostic Osteosarcoma Microenvironment Molecular Subtypes
Source: Front Immunol. 2021 Apr 20;12:623762. doi: 10.3389/fimmu.2021.623762 (PMC8093635; doi:10.3389/fimmu.2021.623762)
Supplement: Supplementary file 1 [file DataSheet_1.pdf]

Table S1: Clinical characteristics of patients in TARGET program osteosarcoma project.

Characteristics	Factor	Group	Count	Percent
Gender	Female	F	39	42.39%
	Male	M	53	57.61%
Diagnosis	Metastatic status at diagnosis	Met	22	23.91%
	Non-metastatic	None	70	76.09%
Overall Survival, OS	>= 1825 days (5 year)	Good	29	31.52%
	< 1825 & >= 730 days (2~5 year)	Medi	32	34.78%
	< 730 days (2 year)	Poor	31	33.70%
Response	Necrosis >= 90% or Stage 3/4	Good	21	22.83%
	Necrosis < 90% or Stage 1/2	Poor	28	30.43%
	No histologic information	None	43	46.74%

Table S2. Clinicopathological characteristics of 47 osteosarcoma patients.

Characteristics	Factor	Count	Percentage (%)
Age (years)	≤ 10	6	12.7
	10 - 18	31	66.0
	18 ≤	10	21.3
Gender	Male	28	59.6
	Female	19	40.4
Enneking	IIB	27	57.4
	III	20	42.6
Lung or Bone metastasis	Yes	21	44.7
	No	26	55.3
Treatment response	PR	15	31.9
	SD	15	31.9
	PD	15	31.9
	NA	2	4.3
